# *In vitro* culture and production of syringin and rutin in *Saussurea involucrata* (Kar. et Kir.) – an endangered medicinal plant

**DOI:** 10.1186/s40529-015-0092-8

**Published:** 2015-05-21

**Authors:** Chao-Lin Kuo, Dinesh-Chandra Agrawal, Hung-Chi Chang, Ya-Ting Chiu, Chu-Peng Huang, Yi-Lin Chen, Shih-Hung Huang, Hsin-Sheng Tsay

**Affiliations:** 1 Department of Chinese Pharmaceutical Sciences and Chinese Medicine Resources, China Medical University, Taichung, Taiwan; 2 Department of Applied Chemistry, Chaoyang University of Technology, Taichung, Taiwan; 3 Department of Golden-Ager Industry Management, Chaoyang University of Technology, Taichung, Taiwan; 4 Department of Biotechnology and Animal Science, National Ilan University, Yilan City, Taiwan; 5 Department of Agronomy, National Chung Hsing University, Taichung, Taiwan

**Keywords:** In vitro culture, Medicinal plant, Rutin, Saussurea involucrata, Snow lotus, Syringin

## Abstract

**Background:**

*Saussurea involucrata* (Kar. et Kir.) commonly known as ‘snow lotus’ or ‘Xue Lian’ is an important plant in the traditional Chinese system of medicine. The plant contains flavonoids such as syringin and rutin. These compounds have been reported to be anti-rheumatic, anti-inflammatory and dilate blood vessels, lower blood pressure, prevent cardiovascular diseases, enhance immunity, and act as anti-aging, anti-cancer, and anti-fatigue agents. The species has become endangered due to the excessive collection of *S. involucrata* plants in the wild, slower plant growth and ecological destruction of natural habitats. There is a severe shortage of plant material, while the market demand is ever increasing. Hence, it is very important to apply tissue culture technique for plant propagation and production of the bioactive compounds of this species.

**Results:**

Multiple shoot induction and proliferation in shoot base explants derived from *in vitro* raised seedlings of *S. involucrata* was achieved on 3/4 strength of Murashige and Skoog’s (MS) basal medium (MSBM) supplemented with 1.0 mg/L^−1^ BA and 1.5 mg/L^−1^ NAA. Rooting was induced in 100 % shoots cultured on 1/2X MSBM supplemented with 1.0 mg/L^−1^ IBA for one week and then transfer to auxin free medium. The plantlets could be acclimatized successfully by sachet technique and established in the greenhouse. Maximum callus induction and proliferation in leaf segments was achieved on 1/2X MSBM supplemented with 0.5 mg/L^−1^ BA, 0.5 mg/L^−1^ NAA, 0.4 % gelrite and on incubation at 20 °C. Container closures had an influence on the quality and quantity of callus and production of the active compounds. The HPLC analysis showed much higher syringin content in *in vitro* shoots and callus as compared to commercially available market crude drug.

**Conclusion:**

The present study describes an *in vitro* culture protocol of *Saussurea involucrata.* The bioactive compounds, syringin and rutin could be produced through tissue culture technique without sacrificing the endangered *Saussurea involucrata* plants in the wild.

**Electronic supplementary material:**

The online version of this article (doi:10.1186/s40529-015-0092-8) contains supplementary material, which is available to authorized users.

## Background

*Saussurea involucrata* (Kar. et Kir.) belonging to the family Asteraceae and commonly known as snow lotus or ‘Xue Lian’ in Chinese mainly grows in the high rocky mountains at 4000 to 4300 m in the Tianshan and A’er Tai areas in Xinjiang province of China (Fu [1992]). The plant has been used in the traditional Chinese system of medicine for over 2000 years for the treatment of many diseases such as rheumatoid arthritis, cough and cold, stomachache, dysmenorrhea, altitude sickness, and has been found to have properties of anti-inflammatory, cardiotonic, abortifacient, anticancer, and antifatigue (Jia et al. [2005]). Considering the importance of the species, recently several researchers carried out investigations with regard to the beneficial effects of *S. involucrata*. In pharmacological studies on *S. involucrata,* anti-inflammatory and anti-nociceptive properties have been reported (Yi et al. [2010]). In another investigation, petroleum ether extract of *S. involucrata* showed high anti-hypoxic activity effective in preventing acute mountain sickness (Ma et al. [2011]). While studying to identify a potential agent for androgen-independent prostate cancer patients and to investigate its biological mechanism as an antineoplastic agent, Way et al. ([2010]) demonstrated that *S. involucrata* effectively inhibited EGFR signaling in human hormone-resistant prostate cancer PC-3 cells. A study on antimetastatic effects on SK-Hep1 human HCC cells found that *S. involucrata* could inhibit cell growth of metastatic cells in dose- and time-dependent manner demonstrating its potential as an anti-tumor agent (Byambaragchaa et al. [2013]). *S. involucrata* produces several bioactive flavonoids that are derived from the phenylpropanoid pathway. A method validation study showed that the LC/MS technique was a powerful analytical tool for detecting trace amounts of the flavonoid compounds in extracts of *S. involucrata* (Xu et al. [2009]). In a separate study, ethyl acetate extract from the aerial parts of *S. involucrata* led to the isolation of three new sesquiterpene lactones having significant anti-inflammatory and cytotoxic activities against A549 cells (Xiao et al. [2011]). Pharmacological properties of syringin and rutin have been studied by several research groups. Syringin has been reported to possess anti-inflammatory and antinociceptive effects (Choi et al. [2004]), hypoglycemic effect (Niu et al. [2008]), antidepressant activity (Kurkin et al. [2006]) and antitumor activity (Zhang et al. [2007]). Rutin has several pharmacological properties including antioxidant (Katsube et al. [2006]), anticarcinogenic, cytoprotective (Janbaz et al. [2002]), anti-platelets (Sheu et al. [2004]), and vasoprotective activities (La Casa et al. [2000]). Moreover, rutin was found to be a neuroprotective agent, and can ameliorate ischemic-reperfusion injury in the heart (Lebeau et al. [2001]), brain (Gupta et al. [2003]) and skeletal muscle (Neumayer et al. [2006]).

Owing to slow plant growth, over exploitation of wild plants, and ecological destruction of the natural habits in recent years, there is a severe shortage of plant material of this species, while the market demand is ever increasing. The wild plant population has diminished to the extent that *S. involucrata* is now considered as an endangered species and has been listed as a national protected wild plant in China (Fu [1992]). Due to these reasons, the isolation of active constituents from the wild plant is a serious constraint. Hence, there is a need to investigate alternative method for plant propagation and isolation of the active compounds. Plant tissue culture system can be an effective means to achieve these objectives. In the present study, Response Surface Methodology (RSM) was used to optimize a suitable medium for *in vitro* culture and callus induction in *S. involucrata.* HPLC analysis of *in vitro* cultures and market crude drug was carried out to estimate the active compounds syringin and rutin.

## Methods

### Plant material

Seeds of *Saussurea involucrata* were provided by Professor C.T. Wei of Department of Biotechnology, China Medical University, Taichung, Taiwan. Seeds were surface sterilized by submerging them into 70 % ethyl alcohol for 30 s followed by 10 % sodium hypochloride for 10 min, and then rinsing them 3 times with sterile double distilled water. Disinfected seeds were inoculated in glass test tubes (measuring 120 mm long, 22 mm dia) containing Murashige and Skoog’s (Murashige and Skoog [1962]) salts and vitamins, hereinafter referred as MS basal medium (MSBM) supplemented with 0.9 % agar and 3 % sucrose. The pH of the medium was adjusted to 5.7 ± 0.1, prior addition of agar and before autoclaving for 15 min under 1.05 kg/cm at 121 °C. All the cultures were incubated at 25 ± 2 °C, under 14/10 h light and dark period and light intensity of 100 μE/m^2^s provided by cool-white fluorescent lights. *In vitro* raised seedlings were used as source material for different experiments in the present study.

### Initiation and proliferation of shoots

**Fig. 1:**
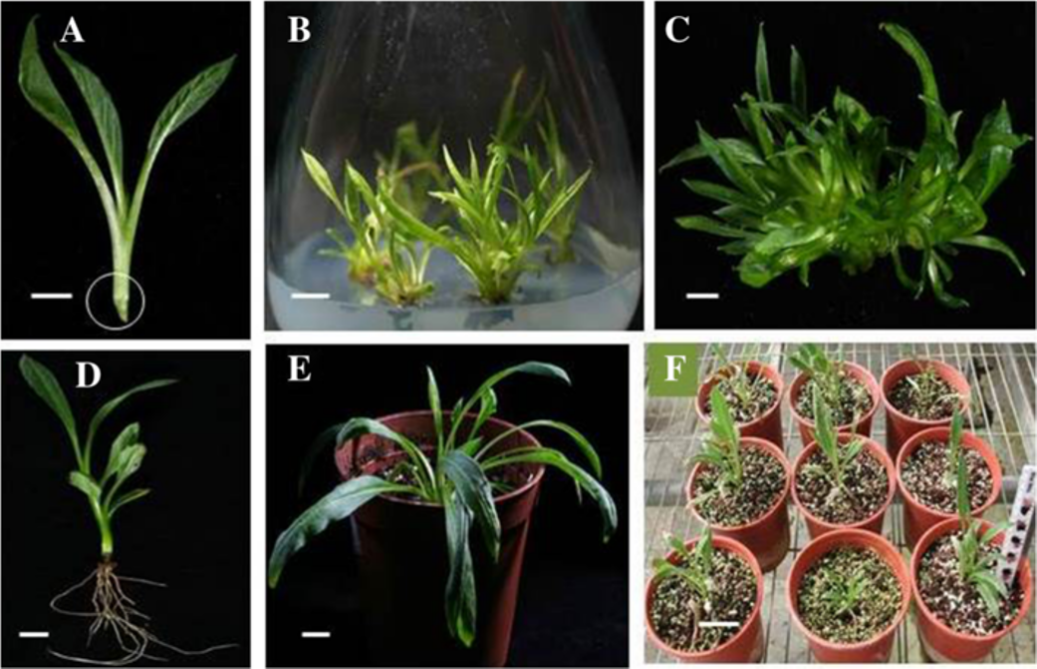
*In vitro* culture of *Saussurea involucrata* Kar. et Kir. **a**: Shoot base explant (shown as in circle); **b**: Multiple shoots; **c**: Proliferation of shoots; **d**: Rooted shoot; **e** and **f**: Tissue culture plants in green house. Scale bar for **a**–**d** = 1 cm; **e** & **f** = 2 cm

In an initial trial, five different basal media (salts and vitamins) viz. MS (Murashige and Skoog, [1962]), WPM - Woody plant medium (Lloyd and McCown [1981]); B5 (Gamborg et al. [1968]); N6 (Chu et al. [1975]) and White ([1963]) without plant growth regulators (PGRs) were tested for initiation of shoots. Since, the best response was observed with MS basal medium, hence all further experiments were carried out with MS alone. Shoot base (Fig. 1a) excised from *in vitro* raised seedlings of *S. involucrata* was used as an explant for shoot initiation. Using RSM, a central composite factorial design was used for media optimization with 3 components viz. MS strength (1X, 3/4X, 1/2X and 1/4X), four concentrations of BA (1.0, 1.5, 2 and 2.5 mg/L^−1^) and NAA (0.5, 1.0 and 1.5 mg/L^−1^). All media were supplemented with 3 % sucrose and pH adjusted to 5.7 ± 0.1 prior to autoclaving. Glass test tube was used as a culture vessel closed with 2 layers of aluminum foil (2AF). After first subculture, explants with initiated shoots were transferred to 650 ml capacity glass orchid flasks for further proliferation. Each culture flask containing 100 ml medium was closed with 4 layers of dispense paper (DP4) and an additional layer of parafilm which was removed after 2 weeks. Both vitrified and normal shoots were cultured as separate sets. Cultures were incubated for total 7 weeks at 25 ± 2 °C, under 14/10 h light and dark period and light intensity of 100 μE/m^2^s provided by cool-white fluorescent lights.

### Rooting of ***in vitro*** shoots

For induction of rooting in *in vitro* shoots, an initial trial was carried out with 3 auxins (IAA, IBA and NAA) individually, at a fixed concentration of 0.5 mgL^−1^. Based on the higher response of rooting on MSBM containing IBA, further experiments were carried out with IBA alone. To optimize the rooting medium, 3 concentrations of IBA (0.1, 0.5 and 1 mgL^−1^), 1/2 strength of MSBM and two concentrations of sucrose (1.5 and 3 %) were tested in a three factorial design. *In vitro* shoots were inoculated on medium with IBA for 1, 2, 3, and 4 weeks and then transferred to medium without IBA for remaining 6, 5,4 and 3 weeks, respectively. All media were gelled with 0.9 % agar. Orchid culture flasks were used as culture vessel and closed with two layers of non-permeable aluminum foil for two weeks, then replaced by 4 layers of dispense paper with an additional layer of parafilm which was removed after 2 weeks. All the cultures were incubated at 25 ± 2 °C, light and dark period of 14/10 h and light intensity of 100 μE/m^2^s provided by cool-white fluorescent lights. Observations like the number of rooted shoots, number of roots and length of roots were recorded after 50 days of incubation.

### Transfer to soil

Rooted shoots were removed from the culture vessel and agar adhering to the roots was removed by gently rinsing with running tap water. Then, these rooted shoots were transferred to plastic pots (15 cm dia. each) containing a mixture of peat soil: perlite: vermiculite (1:1:1) and kept in a greenhouse. Hardening of the plantlets was achieved by following the sachet technique described in our earlier report (Huang et al. [2014]). The plants were irrigated once daily and survival was recorded after two months of transfer to pots.

### Induction and proliferation of callus

Petiole, leaf and root parts of in vitro raised seedlings of *S. involucrata* were used as explants for callus induction. Explants were cultured on 1/2X MSBM supplemented with 2,4-D (0.5, 1.0 mgL − 1), BA (0.5, 1.0, 2.0 mgL − 1), and NAA (0.5 mgL − 1), 3 % sucrose and 0.4 % gelrite. The pH of all media was adjusted to 5.7 ± 0.1. Petri-dishes (9 cm dia), each containing 25 ml of medium were used for callus induction experiment. While, for proliferation experiment, glass test tubes, each containing 10 ml medium were used. Callus (200 mg) was inoculated in each test tube and tubes were closed either with two layers of non-permeable aluminum foil (2AF) or with 3 dispense papers (3DP). Dispense papers had an additional layer of parafilm which was removed after 2 weeks. Observations on callus fresh weight under each treatment was recorded after 4 weeks of incubation. Experiments were carried out to optimize the callus proliferation using factors like temperature (10, 20, 25 °C), and 5 chemical elicitors viz. salicylic acid, abscisic acid, phenylalanine, sodium acetate and jasmonic acid (at a fixed concentration 3 mgL − 1) supplemented individually to 1/2X MSBM. All the cultures were incubated at 25 ± 2 °C (except for temperature experiment), under dark.

### HPLC analysis

HPLC analysis was carried out to quantify syringin and rutin contents in *in vitro* shoots, callus and market crude drug. A reversed-phase symmetry-1 column (250 mm, 4.6X; 5 μm particle size; Waters, USA) was used. The mobile phase was made up from solvent A (methanol) and solvent B (aqueous 0.3 % formic acid in HPLC grade water). The solvent system used was a gradient of aqueous 0.3 % formic acid and methanol. The gradient was as follows: 0 min, A; 3 min, A; 5 min, 15 %, B; 15 min, 30 %, B; 20 min, 30 %, B. The flow rate was 1.0 ml/min and the injection volume was 10 μL. The chromatogram was monitored at 270 nm with a Waters DAD detector. Pure syringin and rutin samples used in the study as standards were procured from Formosa Kingstone Bioproducts International Corporation, Taiwan. Quantification was based on the peak area of the original sample injected. The recovery rate of extraction was evaluated by known quantities of syringin and rutin added to the biomass samples. The accuracy and reproducibility of syringin and rutin measurements were confirmed by analyzing different quantities of the samples.

### Statistical analysis

Software SAS 9.1 was used for statistical analysis. Data were subjected to the least significant difference (LSD) tested at 5 % probability level (*p ≥* 0.05). Each treatment had minimum 30 replicates and all experiments were repeated minimum three times.

## Results and discussion

### Initiation and proliferation of shoots

Shoot initiation response in shoot base explants derived from *in vitro* raised seedlings of *S. involucrata* was very poor. Out of the five basal media tested, the maximum response in 49 % explants with an average 0.85 shoot/explant was observed on 1/2X MSBM after 50 days of incubation. Non-responding shoots turned brown. In the first sub-culture, number of shoots per explant increased to 3.89 in 93.3 % explants however shoots became vitrified (Fig. 1b). In the second subculture, a marked difference was observed between vitrified and normal (non-vitrified) explants and media composition with respect to the number of proliferating shoots. The maximum shoots (13 shoots/per explant) were observed on 3/4X MSBM supplemented with BA (1.0 mg/L^−1^) and NAA (1.5 mg/L^−1^) in case of normal (non-vitrified) explants, while vitrified explants resulted in 4.2 shoots/explant (Fig. 1c). The next best shoot proliferation (11.1 shoots/explant) was achieved on 1/4X MSBM supplemented with 2.0 mg/L^−1^ BA and 0.5 mg/L^−1^ NAA in case of non-vitrified explants. Since, media combinations in the 3-factorial experiment are too many to tabulate, hence, here we have described only the best responses observed with regard to the number of shoots.

In an earlier study, a supplement of BA and NAA was reported to be the most effective growth regulator combination for induction of shoots in leaf explants of *S. involucrata* via organogenesis route (Guo et al. [2007]). They initially recorded 5.2 shoots/explant on BA and NAA containing medium, but this shoot number could be increased to 9.3 by pre-treatment of leaf explants to 4 °C for 5 days. In the present study, a dramatic increase in shoot proliferation in second subculture could be due to culture of shoots in the larger vessels (orchid flasks having 650 ml volume) compared to glass tubes (45 ml volume) used in the first two cultures. Also, culture tube and orchid flask had differential medium content of 10 ml and 100 ml, respectively. Thus, orchid flask had more volume and more medium content compared to the culture tube. Akin to our observation, a positive influence of larger culture vessel on shoot proliferation has been earlier been reported (McClelland and Smith [1990]; Agrawal et al. [1997]; Hazra et al. [2000]). The positive influence of the larger vessel on increased shoot proliferation could be due to the availability of higher amounts of nutrients and a larger area for gaseous exchange.

### Rooting of ***in vitro*** shoots

In an initial trial, out of three auxins (IAA, IBA and NAA) tested at a fixed concentration of 0.5 mg/L^−1^, the best rooting response was observed with IBA. Therefore, in order to further optimize the rooting medium, 3 concentrations of IBA (0.1, 0.5 and 1.0 mgL^−1^) and two concentrations of sucrose (1.5 and 3 %) in the 1/2X MSBM were investigated. The maximum rooting response in 100 % shoots, with an average of 6 roots/explant, and 6.5 cm root length was observed on 1/2X MSBM supplemented with 1.0 mg/L^−1^ IBA and 3 % sucrose (Table 1). This treatment induced healthier roots and shoots compared to all other treatments (Fig. 1d). Medium with 0.1 mg/L^−1^ IBA, though induced rooting in 100 % shoots, however average number of roots per shoot were limited to 3.57 and 2.71 with 1.5 % and 3 % sucrose in the medium, respectively (Table 1). Out of 4 IBA treatments for 1,2,3 and 4 weeks, the maximum rooting response was observed with IBA for 1 week and other three treatments did not show significant differences. Hence, Table 1 contains data pertaining to treatment of IBA for 1 week only. It was observed that shoots incubated in medium with auxin continuously for 50 days induced excessive callusing at the base and roots were shorter in length.

**Table 1:** The influence of IBA and sucrose concentrations on rooting of *in vitro* shoots in *Saussurea involucrata* Kar. et Kir

IBA (mg/L^−1^)*	Sucrose %	% of shoots rooted**	Av. no. of roots/explant**	Av. root length (cm)**	Av. shoot height (cm)**
0.1	1.5	100^a^	3.57^abc^	5.8^a^	4.50^bc^
	3.0	100^a^	2.71^a^	5.6^a^	3.57^ab^
0.5	1.5	79^c^	3.00^ab^	4.3^ab^	4.87^a^
	3.0	93^a^	3.92^abc^	5.7^ab^	4.14^abc^
1.0	1.5	86^ab^	3.33^abc^	4.5^a^	5.91^abc^
	3.0	100^a^	6.00^abc^	6.5^a^	5.07^a^

*1/2X MSBM: Murashige and Skoog ([1962]) basal medium (salts and vitamins), supplemented with 3 % sucrose, 0.9 % agar, pH 5.7 ± 0.1. Data were recorded after 7 weeks of culture. Shoots were cultured on IBA containing medim for 1 week and then transferred to PGR free MSBM containing 1.5 % or 3 % sucrose and 0.9 % agar and further incubation for 6 weeks, respectively

**Each treatment had thirty explants with three replicates. Means followed by the same letter (a,b,c) of a column are not significantly different at 5 % level by least significant difference (LSD) test

Efficient rooting of *in vitro* grown shoots is a pre-requisite for the success of micropropagation. It is a common practice nowadays to employ lower salt concentrations in the culture medium for rooting of *in vitro* shoots. Higher salt levels in the medium have often been found inhibitory to root initiation (George et al. [2008]). In a study on *in vitro* propagation of grapes, it was found that an appropriate salt concentration (1/2X or 1/4X MS) was more important than the sucrose level for induction of rooting in *in vitro* cuttings of grapevine (Harris and Stevenson [1979]). The benefit of low salt medium for root initiation may be due to the need for a low nitrogen level, than for an increased osmotic potential (Harris and Stevenson, [1979]). In an another study, it was demonstrated that microcuttings of several tree fruit rootstocks rooted best on MS salts, and the sucrose concentration in the medium was not critical, though its presence in the medium was essential (Dunstan [1981]). Besides salts and sucrose, plant growth regulators (PGRs), especially, auxins are known to affect both root and shoot growth parameters and play an important role in root development. The success of auxin supply depends on its type, concentration and duration in the culture medium. Different plant species respond differently to auxins for the induction of rooting, hence, it is important to optimize the type, concentration of an auxin and duration for which shoots need to be cultured in the auxin medium. In several cases, a pulse treatment with auxins for a short period has also been sufficient for root induction. While shoots of some species require longer exposure. In the present study, exposure of shoots to IBA containing medium for 1 week was sufficient. Continuous exposure of shoots to auxin medium for 7 weeks induced excessive callus, shorter and unhealthy shoots, an observation similar to our earlier report on *G. davidii* (Chueh et al*.*[2001]). However, in contrast to our results, rooting in shoots of *S. involucrata* was achieved with a continuous exposure to IAA containing medium (Guo et al. [2007]).

### Transfer of plantlets to soil and their survival in the greenhouse

The sachet technique used in our previous report (Huang et al. [2014]) was quite successful in hardening the tissue culture plantlets. Plantlets (100 %) could be hardened and established in the soil (Fig. 1e and f).

### Callus induction and proliferation

**Fig. 2:**
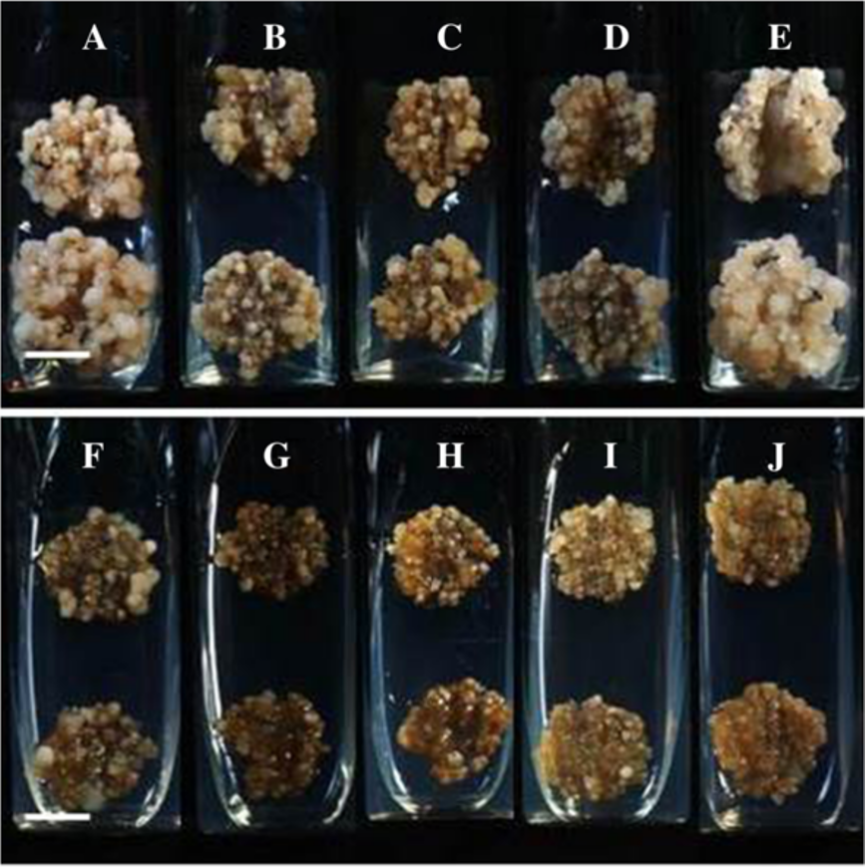
Influence of PGRs and container closure on callus proliferation in *Saussurea involucrata* Kar. et Kir.: **a** and **f**: BA (1.0) + 2,4-D (1.0); **b** and **g**: BA (1.0) + 2,4-D (0.5); **c** and **h**: BA (2.0) + 2,4-D (0.5); **d** and **i**: BA (0.5) + 2,4-D (0.5); **e** and **j**: BA (0.5) + NAA (0.5). PGR conc. - mg/L^−1^; Scale bar for **a**–**j** = 1 cm. Culture medium: 1/2X MSBM, 3 % sucrose, 04 % gelrite. Culture tubes in treatments **a** to **e** were closed with 2 layers of aluminum foil (2AF), while in treatments **f** to **j** were closed with. 3 layers of dispense papers (3DP). Incubation at 20 °C for 4 weeks

Induction of callus in 3 explants viz. leaf, petiole and root of *S. involucrata* varied among the different media compositions. The highest response (80 %) was observed in leaf explants cultured on 1/2X MSBM supplemented with 0.5 or 1.0 mg/L^−1^ BA, 0.5 mg/L^−1^ NAA and 0.4 % Gelrite. In the experiment with the incubation temperature, the maximum callus proliferation was recorded at 20 °C. Sealing of culture vessels with aluminium foil or dispense papers had an influence on the quality and quantity of callus induced (Fig. 2). The maximum callus proliferation (1.036 g) was recorded on medium supplemented with 0.5 mg/L^−1^ BA and 0.5 mg/L^−1^ NAA and culture tubes closed with 3 layers of dispense papers (Table 2). Sealing of culture tubes with 2 layers of aluminum foil resulted in callii compact in texture and darker in color. While, in case of sealing of culture tubes with 3 layers of dispense papers, the callii were friable and fairer. Overall, the quality and quantity of callus proliferated in culture tubes sealed with 3 layers of dispense papers were superior and higher under all treatments compared to culture tubes sealed with 2 layers of aluminum foil (Table 3; Fig. 2). Inclusion of chemical elicitors had a mixed influence on the fresh weight of callus and syringin content (Table 2) All the five elicitors marginally enhanced the callus fresh weight, however, had a negetive influence on syringin content with an exception of jasmonic acid, which mariginally enhanced syringin content in callus (14.788 mg/g dw) compared to control treatment (13.524 mg/g dw). We did not observe any significant effect of any of the five elicitors on rutin content in callus which remained more or less same in all the treatments (Table 2).

**Table 2:** Influence of different elicitors on syringin and rutin content in callus *of Saussurea involucrata* Kar. et Kir

Elicitor*	Fresh weight (g)**	Syringin (mg/g dw)***	Rutin (mg/g dw)
Control	1.31^b^	13.52^a^	0.29
Salicylic acid	1.65^a^	11.30^bc^	0.29
Abscisic acid	1.74^a^	8.75^d^	0.31
Phenylalanine	1.73^a^	10.73^c^	0.37
Sodium acetate	1.58^a^	12.85^ab^	0.32
Jasmonic acid	1.59^a^	14.79^a^	0.32

*Basal medium:1/2X MSBM, BA (0.5 mg/L^−1^), NAA 0.5 (mg/L^−1^), elicitor (3.0 mg/L^−1^), sucrose (3 %), Gelrite (0.4 %) and pH 5.7 ± 0.1. Cultures were incubated at 20 °C. Data were recorded after 4 weeks of culture

**Callus (0.2 g) was taken as inoculum. Each treatment had 50 explants. Four random samples were analyzed in HPLC

***dw: Freeze dry weight; Means followed by the same letter (a,b,c,d) were not significantly different at 5 % level

**Table 3:** Influence of PGRs and container closure on callus proliferation in *Saussurea involucrata* Kar. et Kir

PGR (mg/L^−1^)*			Callus (g)	
2,4-D	BA	NAA	2 Aluminum foils**	3 Dispense papers**
0	0.5	0.5	0.57^b^	1.04^a^
0.5	0.5	0	0.32^e^	0.64^bc^
1.0	1.0	0	0.46^c^	0.74^b^
0.5	1.0	0	0.44^dc^	0.63^bc^
0.5	2.0	0	0.38^de^	0.52^c^

*Basal medium: 1/2X MSBM, 3 % sucrose, 0.4 % Gelrite and pH 5.7 ± 0.1. Data were recorded after 4 weeks of culture

Each treatment had 30 explants in three replicates. Cultures were incubated at 20 °C. Callus (0.2 g) was taken as the initial inoculum

**Means followed by the same letter (a,b,c,d,e) in a column were not significantly different at the % level by LSD test

The physical and chemical micro-environments of culture containers can influence growth rate and other physiological and morphological characteristics of plants developed under *in vitro* conditions (Walker et al. [1988]). It has been reported that the type of closure affects gaseous exchange, availability of water, micronutrients, and balance of hormones in the culture container (Kataeva et al. [1991]; Lai et al. [2005]; Chen et al. [2006a]; Tsay et al. [2006]). The head space of culture vessels with low ventilation, accumulates various gaseous compounds like ethylene and carbon dioxide (Zobayed et al. [2001]; Lai et al. [2005]). These undesirable compounds can alter biochemical responses and leaf development of *in vitro* cultured plants (Pierik et al. [2007]) and also affect enzymes involved in oxidative activities (Synková and Pospíšilová [2002]). Some closures cause restriction of gaseous exchange between the container atmosphere and the outside environment (Buddendorf-Joosten and Woltering [1994]), which can result in poor aeration and hyperhydric condition of cultures. Also, Different species show different requirement with respect to container closures. Hence, it is important to optimize a closure type in a micropropagation protocol of a particular plant species. In our laboratory, aluminum foil and dispense paper have been used successfully to improve ventilation for cultures of *Scrophularia* (Chen et al. [2006a]), and *Bupleurum* (Chen et al. [2006b]). It was observed that aluminum foil, a less air permeable material for first 4 weeks, followed by more air permeable dispense papers for the next 4 weeks was an adequate ventilation treatment for optimum root/shoot growth and subsequent survival of *G.* s*cabra* plantlets (Huang et al. [2014]). In the present study also a combination of foil and dispense paper was found to be a favorable combination for callus proliferation in *S. involucrata.* Similar to our results, a selective influence of chemical elicitors on the enhancement of secondary plant metabolites in callus has been reported earlier (Bulgakov et al. [2002]). It was found that salicylic acid and methyl jasmonate had a positive influence on anthraquinone accumulation in both transgenic and non-transgenic calluses of *Rubia cordifolia* but ethephon did not (Bulgakov et al. [2002]).

### HPLC analysis

HPLC analysis of *in vitro* shoots, callus and market crude drug revealed marked differences in syringin content in these materials (Table 4). The highest syringin content (13.52 mg/g dw) was observed in callus followed by *in vitro* shoots (8.57 mg/g dw) compared to market crude drug (0.95 mg/g dw). There was a marginal difference in rutin content in all the three materials. However, the positive outcome of the present study is that production of both syringin and rutin was achieved in an *in vitro* culture system.

**Table 4:** HPLC analysis: Syringin and rutin conents in different materials of *Saussurea involucrata* Kar. et Kir

Plant material	Syringin (mg/g dw)*	Rutin (mg/g dw)*
Tissue culture shoots	8.37	0.34
Callus	13.52	0.29
Market crude drug	0.95	0.24

*dw: Freeze dry weight

## Conclusions

An *in vitro* culture protocol for *S. involucrata* has been developed using Response Surface Methodology (RSM). Callus induction and proliferation could be achieved by *in vitro* leaf culture method. HPLC analysis results showed that syringin content was much higher both in callus and *in vitro* shoots compared to crude drug available in the market. Between the *in vitro* shoots and callus, the latter showed a higher syringin accumulation and could be a method of choice for the production of syringin and rutin. The present study demonstrates the usefulness of a tissue culture system with regard to production of the active compounds (syringin and rutin) round the year without the destruction of wild *S. involucrata* plants which are already under endangered category.

## Authors’ original submitted files for images

Below are the links to the authors’ original submitted files for images. Authors’ original file for figure 1 Authors’ original file for figure 2

## Abbreviations

AF: Aluminum foil
BA: 6-Benzylaminopurine
DP: Dispense paper
HPLC: High- performance liquid chromatography
IAA: Indole-3-acetic acid
IBA: Indole-3-butyric acid
MS: Murashige and Skoog
MSBM: Muruashige & Skoog’s basal medium (salts and vitamins)
NAA: 1-Naphthalene acetic acid
PGR: Plant growth regulator
RSM: Response surface methodology
WPM: Woody plant medium
