# Supplementary figures and images for: In vitro culture and production of syringin and rutin in Saussurea involucrata (Kar. et Kir.) – an endangered medicinal plant
Source: Bot Stud. 2015 May 21;56:12. doi: 10.1186/s40529-015-0092-8 (PMC5430372; doi:10.1186/s40529-015-0092-8)

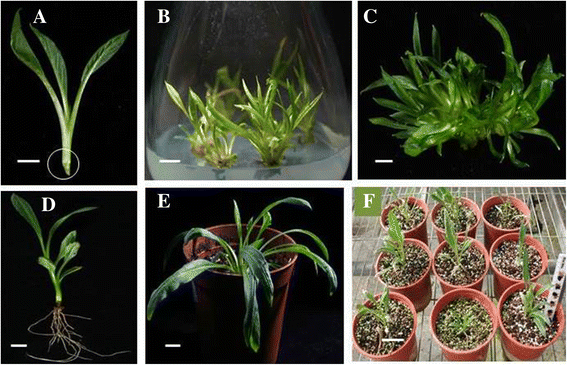

Supplement: Supplementary file 1 — Authors’ original file for figure 1 [file 40529_2015_92_MOESM1_ESM.gif]

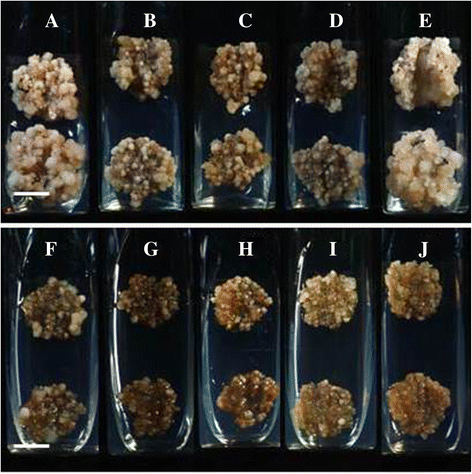

Supplement: Supplementary file 2 — Authors’ original file for figure 2 [file 40529_2015_92_MOESM2_ESM.gif]
